# Variability in blood lipids affects the neutrophil to lymphocyte ratio in patients undergoing elective percutaneous coronary intervention: a retrospective study

**DOI:** 10.1186/s12944-020-01304-9

**Published:** 2020-06-03

**Authors:** Liding Zhao, Tian Xu, Ya Li, Yi Luan, Qingbo Lv, Guosheng Fu, Wenbin Zhang

**Affiliations:** 1 Department of Cardiovascular Diseases, Sir Run Run Shaw Hospital, College of Medicine, Zhejiang University, No 3 East of Qinchun Road, Hangzhou, Zhejiang 310000 People’s Republic of China; 2 Key Laboratory of Cardiovascular Intervention and Regenerative Medicine of Zhejiang Province, Hangzhou, China

**Keywords:** Variability, High-density lipoprotein cholesterol, Low-density lipoprotein cholesterol, Neutrophil to lymphocyte ratio, Percutaneous coronary intervention, Atherosclerosis

## Abstract

**Background:**

Atherosclerosis is associated with chronic inflammation and lipid metabolism. The neutrophil to lymphocyte ratio (NLR) as an indicator of inflammation has been confirmed to be associated with cardiovascular disease prognosis. However, few studies have explored the effects of blood lipid variability on NLR. The aim of this study was to explore the relationship between variability in blood lipid levels and NLR.

**Methods:**

The association between variability in blood lipids and NLR was assessed with both univariate and multivariate linear regression. Multivariate linear regression was also performed for a subgroup analysis.

**Results:**

The variability of high-density lipoprotein cholesterol (HDL-C) (regression coefficients [β] 4.008, standard error (SE) 0.503, *P*-value< 0.001) and low-density lipoprotein cholesterol (LDL-C) ([β] 0.626, SE 0.164, *P*-value< 0.001) were risk factors for the NLR value, although baseline LDL-C and HDL-C were not risk factors for NLR values. Variability of HDL-C ([β] 4.328, SE 0.578, *P*-value< 0.001) and LDL-C ([β] 0.660, SE 0.183, *P*-value< 0.001) were risk factors for NLR variability. Subgroup analysis demonstrated that the relationship between variability of LDL-C and NLR was consistent with the trend of the total sample for those with diabetes mellitus, controlled blood lipid, statins, atorvastatin. The relationship between the variability of HDL-C and NLR was consistent with the trend of the total sample in all subgroups.

**Conclusion:**

The variability of HDL-C and LDL-C are risk factors for the value and variability of NLR, while the relationship between variability of HDL-C and NLR is more stable than the variability of LDL-C in the subgroup analysis, which provides a new perspective for controlling inflammation in patients undergoing PCI.

## Background

Atherosclerosis, which is associated with lipid metabolism, is a common disease characterized by lipid deposition of the arterial intimal layer as well as formation of atherosclerotic plaques [1, 2]. Atherosclerosis accounts for elevated morbidity and mortality worldwide [3]. It has recently been found that blood lipid theory is key for the development of atherosclerosis and that low-density lipoprotein cholesterol (LDL-C) plays a key role in this [1]. Indeed, elevated levels of triglyceride (TG) and LDL-C in serum and a decrease in high-density lipoprotein cholesterol (HDL-C) are considered the lipid triads of atherosclerosis [4].

Atherosclerosis is also an inflammatory process that responds to various risk factors [5]. Chronic inflammation of the arterial wall is important for the development of atherosclerosis [6]. As a cost-effective, readily available indicator of inflammation, the neutrophil to lymphocyte ratio (NLR) is predictive of patients’ prognosis in the metastatic urothelial cancer (mUC) following immune-checkpoint inhibitors [7] and shows prognostic value for predicting the 30-day mortality rate and 3-month readmission rate of community-acquired pneumonia (CAP) patients [8]. More importantly, NLR has been shown to be associated with the severity and prognosis of many cardiovascular diseases, including coronary atherosclerosis [9, 10]. Authors of previous studies have confirmed that NLR is a predictor of in-hospital major adverse cardiovascular events [11] and long-term prognosis [12] in patients after PCI. Even in the typical white blood cell count range, a higher NLR is associated with atherosclerotic events [13]. In both healthy people [14] and coronary artery disease (CAD) patients [15], low HDL-C has been shown to correlate with the rise of NLR, although whether the variability of lipid protein has any effect on NLR remains unclear.

The aim of this study was to investigate the relationship of blood lipids as well as their variability with NLR in patients who were undergoing elective percutaneous coronary intervention (PCI).

## Methods

### Population and procedures

This single center, observational, retrospective study analyzed data from 4445 patients consecutively admitted to the Sir Run Run Shaw Hospital, Zhejiang University in China between January 2009 and April 2019. Inclusion criteria were as follows: (1) patients must have undergone elective percutaneous coronary intervention; (2) NLR and lipid values during follow-up such as total cholesterol (TC), TG, LDL-C, HDL-C were available; (3) patients were followed up three times or more in the outpatient clinic within 1 year following PCI.

Participants were excluded if their C-reactive protein (CRP)>10 mg/L, their white blood cell count (WBC)>10 × 10^9/L, they had congenital heart disease, valvular heart disease, heart failure, peripheral arterial disease, severe renal or hepatic dysfunction, hematological disorders, history of malignancy, acute or chronic infection.

All PCI procedures were carried out by experienced interventional cardiologists using the femoral or radial artery approach, as recommended by current guidelines [16]. Blood samples for baseline information were collected 24 h before PCI. Three or more follow-ups were carried out in 1 year for patients who have undergone PCI. After an overnight fast, blood samples were taken by antecubital vein puncture to measure routinely evaluated laboratory values. Total leucocyte count and its subtypes, including neutrophil and lymphocyte, as well as monocyte and platelet count were analyzed using an automated blood cell counter. Lipid values such as total cholesterol (TC), triglycerides (TG), low-density lipoprotein cholesterol (LDL-C), high-density lipoprotein cholesterol (HDL-C), and very low-density lipoprotein (VLDL) were also measured by a blood chemistry analyzer (Hitachi 747; Hitachi, Tokyo, Japan). The study was given approval by the Ethics Committee of Sir Run Run Shaw Hospital of Zhejiang University.

### Definitions

Patients’ medical records from when they were hospitalized were used as baseline indicators and blood samples for baseline information were collected 24 h before PCI. HDL-C (STDEV), LDL-C (STDEV), TG (STDEV) and TC (STDEV) refer to the variability of HDL-C, LDL-C, TG and TC, respectively. These were expressed as standard deviations, calculated from follow-up results obtained in 1 year for patients who have undergone PCI. The value of NLR was the mean of the follow-up results. Lipid control was defined as LDL-C<1.8 mmol/L at each follow-up measurement. A total of 3118 patients had their blood lipids controlled. Smoking was defined as currently smoking or stopping less than one month ago. Heart failure was defined by EF < 40% or NT-pro BNP > 2000 pg/ml.

### Statistical analysis

Statistical analysis was performed using SPSS software version 22.0 (SPSS Inc., Chicago, IL, USA). Continuous variables were non-normally distributed and presented as medians (25–75%). Categorical variables were represented as n (%). Univariate analysis and multivariate regression analysis for each factor were performed by linear regression analysis. In the subgroup analysis, multivariate linear regressions were performed on patients with or without diabetes mellitus, with controlled or uncontrolled blood lipid, who were taking statin with ezetimibe or not, and who were taking rosuvastatin or atorvastatin. All reported *P*-values were two-sided, and *P*-values of < 0.05 were considered statistically significant.

## Results

### Baseline characteristics

In present study, 4445 patients who had undergone elective PCI at the Sir Run Run Shaw Hospital between January 2009 and April 2019 were included, according to the inclusion and exclusion criteria. Participants’ median age was 64 (58–71), 71.9% were men, 63.4% had hypertension and 25.6% had diabetes mellitus. Demographic information, laboratory examination and baseline medication can be seen in Table 1.

**Table 1:** Baseline characteristics of the sample

	Total samples (*n* = 4445)
**Demographic information**	
Age (Years)	64.00 (58.00–71.00)
Man, N (%)	3198 (71.9%)
BMI	24.73 (22.64–26.10)
Current smoking, N (%)	1131 (25.4%)
Diabetes, N (%)	1139 (25.6%)
Hypertension, N (%)	2817 (63.4%)
Previous MI, N (%)	97 (2.2%)
Previous PCI, N (%)	217 (4.9%)
Stable angina pectoris	953 (21.4%)
**Laboratory examination**	
NLR	2.64 (1.94–3.95)
WBC (×10^9/L)	6.60 (5.40–8.30)
PLT (×10^9/L)	182.00 (149.00–219.63)
CRP (mg/L)	1.70 (0.70–5.00)
LDL-C (mmol/L)	2.22 (1.68–2.93)
HDL-C (mmol/L)	1.00 (0.84–1.20)
Lipoprotein (mmol/L)	15.2 (7.78–33.20)
Total cholesterol (mmol/L)	4.19 (3.47–5.01)
Triglyceride (mmol/L)	1.43 (1.03–2.04)
VLDL-C (mmol/L)	0.62 (0.38–0.93)
Creatinine (umol/L)	75.00 (64.00–88.40)
Uric acid (umol/L)	356.00 (296.00–424.00)
LDL-C (STDEV)	0.46 (0.26–0.74)
HDL-C (STDEV)	0.13 (0.08–0.19)
Total cholesterol (STDEV)	0.58 (0.35–0.92)
Triglyceride (STDEV)	0.33 (0.19–0.56)
NLR (mean)	2.97 (2.27–4.11)
CRP (mean, mg/L)	1.53 (0.80–2.87)
**Baseline medication**	
ACEI, N (%)	1136 (25.6%)
ARB, N (%)	1558 (35.1%)
Beta blocker, N (%)	2600 (58.5%)
CCB, N (%)	1319 (29.7%)
Antiplatelet drugs	
Aspirin, N (%)	4284 (96.4%)
Clopidogrel, N (%)	3602 (81.0%)
Ticagrelor, N (%)	749 (16.9%)
Ezetimibe, N (%)	853 (19.2%)
Statin, N (%)	4377 (98.5%)
Atorvastatin, N (%)	2687 (60.4%)
Rosuvastatin, N (%)	1559 (35.1%)
Pravastatin, N (%)	23 (0.5%)
Simvastatin, N (%)	58 (1.3%)
Intensive statin treatment, N (%)	564 (12.7%)

Values are expressed as median (25–75%) or n (%) unless otherwise indicated. STDEV indicates the standard deviation calculated from the follow-up results, mean is the average of the results from the follow-up; *NLR* neutrophil to lymphocyte ratio, *WBC* white blood cell, *PLT* platelet, *CRP* C-creative protein, *BMI* Body Mass Index, *LDL-C* low-density lipoprotein cholesterol, *HDL-C* high-density lipoprotein cholesterol, *VLDL-C* very low-density lipoprotein cholesterol, *ACEI* Angiotensin-Converting Enzyme Inhibitors, *ARB* angiotensin receptor blocker, *CCB* calcium channel blocker

### Results of univariate and multivariate linear regression for the mean of NLR

The univariate analysis suggested that age, gender, diabetes, hypertension, types of statins, HDL-C (STDEV), CRP (mean), LDL-C (mean), creatinine and uric acid were all risk factors for the mean value of NLR during follow-up. In contrast, ezetimibe, baseline TC, baseline HDL-C and HDL-C (mean) were protective factors for the mean value of NLR (see Table 2).

**Table 2:** Results of univariate and multivariate linear regression for the mean of NLR

Variable	Univariate regression			Multiple Regression		
	β	SE	p	β	SE	p
Age	0.047	0.004	**< 0.001**	0.035	0.004	**< 0.001**
Gender	0.366	0.092	**< 0.001**	0.491	0.098	**< 0.001**
Current smoking	−0.029	0.048	0.547			
Diabetes	0.278	0.094	**0.003**	0.054	0.094	0.570
Hypertension	0.286	0.086	**0.001**	0.109	0.087	0.212
Types of statins	0.317	0.051	**< 0.001**	0.350	0.053	**< 0.001**
Intensive statin treatment	−0.146	0.122	0.232			
Ezetimibe	−0.252	0.106	**0.017**	−0.032	0.109	0.772
Baseline TC	−0.109	0.033	**0.001**	−0.182	0.050	**< 0.001**
Baseline LDL-C	−0.019	0.042	0.657			
Baseline HDL-C	−0.589	0.144	**< 0.001**	−0.266	0.162	0.101
LDL-C (STDEV)	0.070	0.112	0.531	0.626	0.164	**< 0.001**
HDL-C (STDEV)	4.648	0.048	**< 0.001**	4.008	0.503	**< 0.001**
TC (STDEV)	0.069	0.089	0.438			
TG (STDEV)	−0.079	0.058	0.170			
CRP (mean)	0.400	0.025	**< 0.001**	0.326	0.026	**< 0.001**
HDL-C (mean)	−1.903	0.162	**< 0.001**	−3.356	0.334	**< 0.001**
LDL-C (mean)	0.180	0.069	**0.009**	0.560	0.163	**0.001**
Creatinine	0.006	0.001	**< 0.001**	0.004	0.001	**< 0.001**
Uric acid	0.001	< 0.001	**0.006**	−0.001	< 0.001	0.087

β indicates regression coefficients, *SE* standard error, *TC* total cholesterol, *TG* triglyceride. Other abbreviations as in Table 1. Level of significance was accepted at *P* < 0.05, and highlighted in bold

Once the univariate analysis was corrected to allow for confounding factors, results from the multivariable logistic regression analysis showed that age, gender, types of statins, HDL-C (STDEV), LDL-C (STDEV), LDL-C (mean), CRP (mean) and creatinine were all risk factors for the mean value of NLR. Baseline TC and HDL-C (mean) were protective factors for the mean value of NLR (see Table 2).

**Fig. 1:**
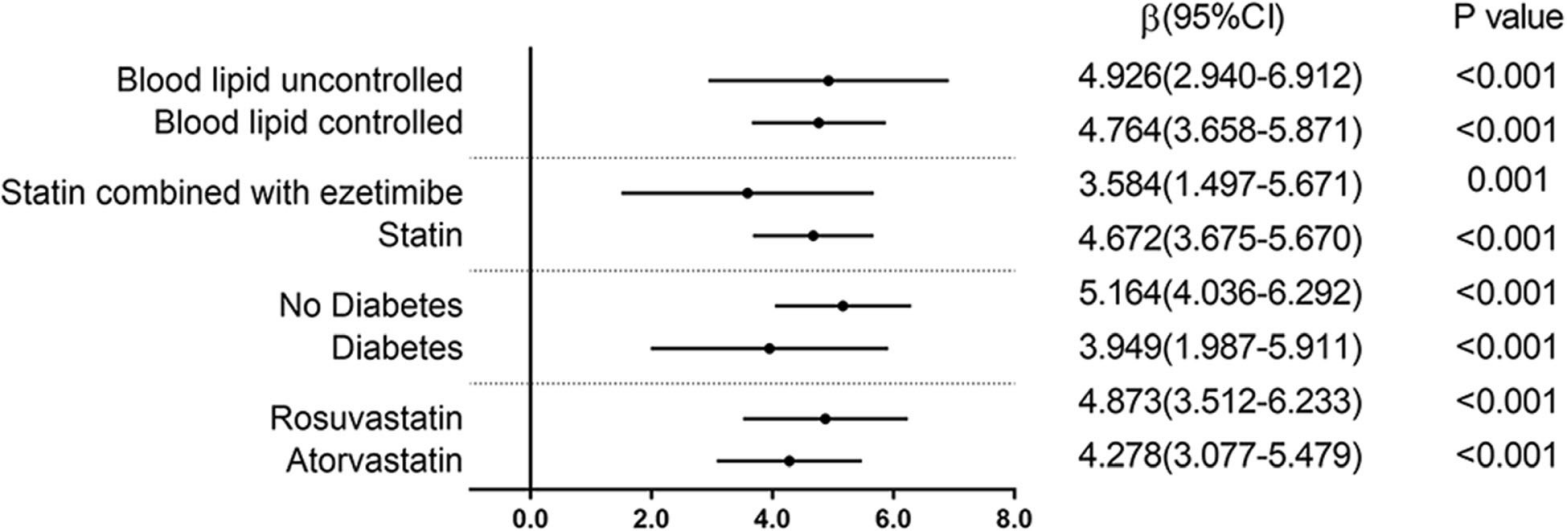
Subgroup analysis of NLR and variability of HDL-C. Multivariate linear regression results for the variability of HDL-C and NLR. β indicates regression coefficients; CI confidence intervals

For HDL-C (STDEV), multivariate linear regression analysis results for each subgroup are stable, showing that HDL-C (STDEV) is a risk factor for the mean value of NLR during the follow-up (see Fig. 1).

**Fig. 2:**
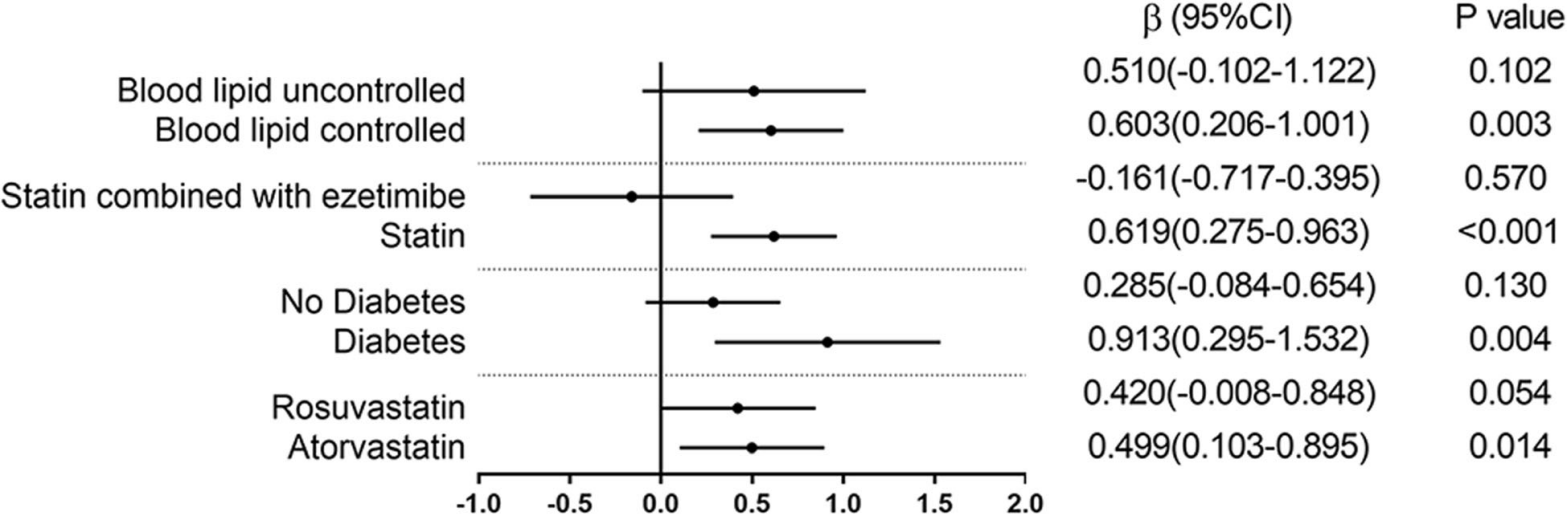
Subgroup analysis of NLR and variability of LDL-C. Multivariate linear regression results for the variability of LDL-C and NLR. β indicates regression coefficients; CI confidence intervals

 In contrast, the relationship between LDL-C (STDEV) and the mean value of NLR was consistent across patients with blood lipid controlled (regression coefficients [β] 0.603, [95% CI] 0.204–1.001, *P*-value = 0.003), diabetes ([β] 0.913, [95% CI] 0.295–1.532, *P*-value = 0.004), those who were taking statins ([β] 0.0.619, [95% CI] 0.275–0.963, *P*-value< 0.001) and those taking atorvastatin ([β] 0.499, [95% CI] 0.103–0.895, *P*-value = 0.014) (see Fig. 2).

### Results of univariate and multivariate linear regression for the variability in NLR

The univariate analysis suggested that age, gender, types of statins, HDL-C (STDEV), CRP (mean) and creatinine were all risk factors for NLR variability during follow-up. In contrast, baseline TC, HDL-C (mean) and baseline HDL-C were protective factors for NLR variability (see Table 3).

**Table 3:** Results of univariate and multivariate linear regression for variability in NLR

Variable	Univariate regression			Multiple Regression		
	β	SE	p	β	SE	p
Age	0.034	0.004	**< 0.001**	0.024	0.005	**< 0.001**
Gender	0.303	0.102	**0.003**	0.320	0.108	**0.003**
Current smoking	−0.057	0.054	0.289			
Diabetes	0.125	0.105	0.232			
Hypertension	0.099	0.095	0.298			
Types of statins	0.261	0.056	**< 0.001**	0.304	0.061	**< 0.001**
Intensive statin treatment	−0.143	0.136	0.293			
Ezetimibe	−0.214	0.117	0.068			
Baseline TC	−0.079	0.036	**0.029**	−0.175	0.055	**0.002**
Baseline LDL-C	0.008	0.047	0.859			
Baseline HDL-C	−0.553	0.160	**0.001**	−0.381	0.185	**0.040**
LDL-C (STDEV)	0.194	0.124	0.120	0.660	0.183	**< 0.001**
HDL-C (STDEV)/	4.680	0.533	**< 0.001**	4.328	0.578	**< 0.001**
TC (STDEV)	0.168	0.099	0.089			
TG (STDEV)	0.003	0.064	0.964			
CRP (mean)	0.321	0.029	**< 0.001**	0.256	0.030	**< 0.001**
HDL-C (mean)	−1.615	0.181	**< 0.001**	−3.472	0.358	**< 0.001**
LDL-C (mean)	0.097	0.076	0.205	0.062	0.112	0.578
Creatinine	0.005	0.001	**< 0.001**	0.003	0.001	**< 0.001**
Uric acid	0.001	< 0.001	0.093			

β indicates regression coefficients, *SE* standard error, *TC* total cholesterol, *TG* triglyceride. Other abbreviations as in Table 1. Level of significance was accepted at *P* < 0.05, and highlighted in bold

After correction for the confounding factors screened from the univariate analysis, the multivariable linear regression analysis revealed that age, gender, types of statins, HDL-C (STDEV), LDL-C (STDEV), CRP (mean) and creatinine were risk factors for NLR variability. Baseline TC, HDL-C (mean) and baseline HDL-C were protective factors for the variability in NLR (see Table 3).

**Fig. 3:**
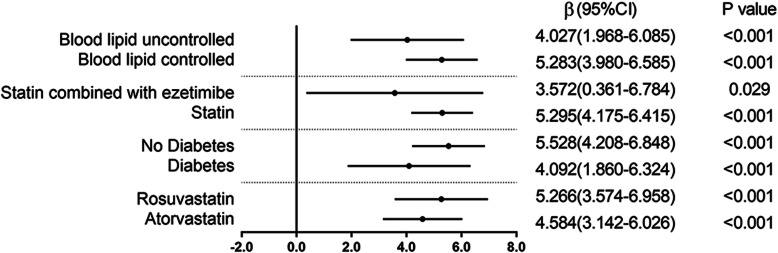
Subgroup analysis of variability in NLR and variability of HDL-C. Multivariate linear regression results for the variability of HDL-C and variability in NLR. β indicates regression coefficients; CI confidence intervals

For HDL-C (STDEV), multivariate linear regression analysis results for each subgroup are stable, suggesting that HDL-C (STDEV) is a risk factor for NLR variability during the follow-up (see Fig. 3).

**Fig. 4:**
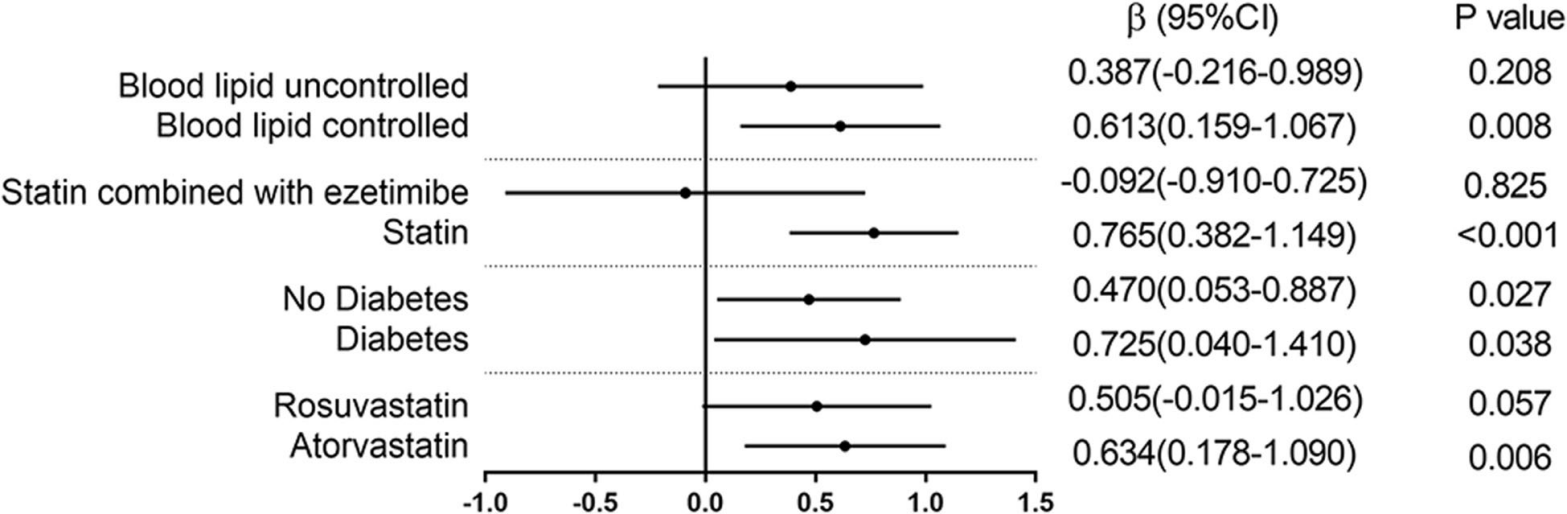
Subgroup analysis of variability in NLR and variability of LDL-C. Multivariate linear regression results for the variability of LDL-C and variability in NLR. β indicates regression coefficients; CI confidence intervals

On the other hand, the relationship between LDL-C (STDEV) and NLR variability was consistent across the patients with controlled blood lipid ([β] 0.613, [95% CI] 0.159–1.067, *P*-value = 0.008), diabetes ([β] 0.725, [95% CI] 0.040–1.410, *P*-value = 0.0038), those with no diabetes ([β] 0.470, [95% CI] 0.053–0.887, *P*-value = 0.027), those taking statins ([β] 0.765, [95% CI] 0.382–1.149, *P*-value< 0.001), and those taking atorvastatin ([β] 0.634, [95% CI] 0.178–1.090, *P*-value = 0.006) (see Fig. 4).

## Discussion

The main findings of the current study are as follows: (1) variability of HDL-C and LDL-C were risk factors for the value and variability in NLR; (2) the relationship between variability of HDL-C and NLR was consistent for each subgroup analysis (those with or without diabetes mellitus, controlled or uncontrolled blood lipid, taking statin with ezetimibe or not, and taking rosuvastatin or atorvastatin). The relationship between variability of LDL-C and NLR was also confirmed in patients with controlled blood lipid, diabetes, those taking statin and those taking atorvastatin. Subgroup analysis results suggest that the linear relationship between HDL-C and NLR is more stable than for LDL-C.

Blood lipid levels play a crucial role in the process of atherosclerosis [1, 17]. HDL-C is an independent predictor of coronary heart disease risk [18]. Authors of four large studies have concluded that each increase of 1 mg per deciliter (0.03 mmol per liter) in HDL-C is associated with a decrease of 2 to 3% in future coronary heart disease risk [19]. Low-density lipoprotein has important physiological effects as a carrier for transporting cholesterol to peripheral tissues, although its elevated level is associated with an increased risk of cardiovascular disease [1, 20, 21]. NLR can be easily calculated from differential WBC counts, which are widely available and routinely performed. These counts provide physicians with important information regarding not only the prognosis of mUC [7] and CAP [8] patients, but also with additional risk stratification beyond conventional risk scores such as predicting mortality in cardiovascular diseases [6]. Increased NLR values may indicate subclinical inflammation [22]. In addition, NLR is associated with pro-inflammatory mediators (high sensitive CRP, tumor necrosis factor-α) and progressive subclinical atherogenesis [23]. A high NLR is a predictor of atherosclerosis progression [10, 24, 25] and long-term prognosis in patients after PCI [12]. As a protective factor for blood vessels, HDL-C has been observed in both healthy people [14] and CAD patients [15] as associated with NLR levels at lower levels. However, few authors have focused on the relationship between variability in blood lipids and NLR.

Visit-to-visit LDL-C variability was found to be an independent predictor of cardiovascular events for patients with stable coronary artery disease in the TNT trial [26], while Boey et al. confirmed that visit-to-visit LDL-C and HDL-C variability are associated with occurrence of a MACE at 5 year follow-up following STEMI [27]. Results of the current study further support the clinical relevance of LDL-C and HDL-C variability by showing that LDL-C and HDL-C variability are risk factors for NLR. In contrast to Boey et al., the focus of this study is on NLR rather than clinical outcomes. The current study sample was Asian rather than Caucasian patients who were followed-up for 1 year following PCI. In addition, by confirming the variability in HDL-C and LDL-C can affect NLR variability, the clinical connection between blood lipid and NLR was further explored. The results of the current study may have certain reference significance for lipid-lowering therapy in patients at this time.

The exact mechanism for increased variability in LDL-C, HDL-C and an increased risk of NLR remains unknown. However, several hypotheses have been suggested. Statins primarily (although not exclusively) stabilize plaque [28] through a cholesterol-dependent mechanism, thereby reducing plaque cholesterol levels [29]. In turn, lipid reduction inhibits inflammation as well as reducing collagen hydrolyzing activity and thrombotic potential. LDL-C variability may result in instability of the vessel wall due to damage to cholesterol-dependent plaque stabilization mechanisms [26], thus increasing the likelihood of plaque vulnerability and rupture, although most ruptures do not cause clinic events. Conversely, high HDL-C variability may lead to plaque instability by impairing cholesterol outflows in surrounding tissues and macrophages, thus increasing the risk of damage to the vessel wall and inducing inflammation [30]. Additionally, LDL particles in circulation can penetrate the endothelium of the arterial wall and be oxidized, promoting inflammation and causing endothelial damage [31]. Neutrophils indicate systemic inflammatory states, while lymphocytes suggest fibrotic hyperplasia and homeostasis of overall inflammation. Both of these respond to inflammation caused by arterial plaque [2, 32, 33]. Further to this, vasculogenesis, a process involved in cardiovascular injury, initiates various chronic adaptive processes including elevation of circulating neutrophils, which will further damage vascular endothelial cells through processes such as inflammatory reactions and oxidative stress [34], partially explaining this study’s results. Interestingly, the use of subgroup analysis in this study showed that the relationship between the variability of HDL-C and NLR is more stable than the variability of LDL-C, which deserves further investigation. In clinical practice, there is no effective treatment method for controlling NLR. It was found that blood lipid variability is an independent risk factor for NLR, suggesting that the control of blood lipid variability can affect NLR, thereby improving the prognosis of patients undergoing PCI.

### Limitations

First, as a single center, retrospective observational study, residual confounding or selection bias cannot be excluded, which is inherent to any retrospective study. Second, patients’ statin dose was not taken as a factor of inquiry, which may affect blood lipid variability. Third, factors that may affect NLR, such as hypertension and diabetes, were not ruled out, but instead were used as corrective factors in the multivariate regression. Last but not least, information on patients’ interleukin-6, which can reflect the inflammatory status in vivo, was not collected. Additionally, there was no focus on the relationship of CRP, another indicator of inflammation, with blood lipid variability, but this was included in multivariate regression.

## Conclusion

The variability of HDL-C and LDL-C are risk factors for NLR in patients who have undergone elective percutaneous coronary intervention, while the relationship between the variability of HDL-C and NLR is more stable than the variability of LDL-C.

## Data Availability

The datasets used and/or analyzed during the current study are available from the corresponding author on reasonable request.

## Abbreviations

NLR: Neutrophil to lymphocyte ratio
LDL-C: Low-density lipoprotein cholesterol
HDL-C: High-density lipoprotein cholesterol
TG: Triglyceride
TC: Total cholesterol
CAD: Coronary artery disease
PCI: Percutaneous coronary intervention
CRP: C-reactive protein
WBC: White blood cell count
VLDL: Very low-density lipoprotein
CI: Confidence intervals
